# Improvement of Erectile Function After Switching From Valsartan to Olmesartan Within a Fixed‐Dose Antihypertensive Combination: A Case Report

**DOI:** 10.1002/ccr3.73718

**Published:** 2026-10-16

**Authors:** Mario Ćurković, Nika Ćurković

**Affiliations:** ^1^ Osijek ‐ Baranja County Health Center Osijek Croatia; ^2^ Medical Faculty Osijek University Josip Juraj Strossmayer Osijek Osijek Croatia; ^3^ Health Center Zagreb‐Center Zagreb Croatia

**Keywords:** eNOS, erectile dysfunction, hypertension, IIEF‐5, Naranjo algorithm, olmesartan, valsartan

## Abstract

Intra‐class differences may exist between ARBs regarding tissue‐specific outcomes. In this case, valsartan was probably associated with erectile dysfunction (Naranjo score 7). Switching to olmesartan was followed by resolutions of symptoms. An intra‐class switch may be considered in selected patients with suspected drug‐associated erectile dysfunction after evaluating other vascular, metabolic, or lifestyle contributions. Clinicians should remain cautious, as concurrent treatment modifications can confound definitive causal attributions.

## 1 Introduction

Erectile dysfunction (ED) is a major health issue with a high overall prevalence, ranging from 3% to over 50% according to current data, and shows a strong correlation with patient age [1, 2]. Clinical research highlights a direct association between ED and vascular risk factors such as hypertension and diabetes mellitus, as well as with the adverse effects of the pharmacotherapy used to treat these conditions [1]. ED is now considered a potential predictor of increased cardiovascular mortality and risk of dementia, reflecting a potentially shared vascular pathophysiology among these conditions [2]. The clinical presentation in younger patients is particularly important, as ED can be the initial manifestation of systemic vascular disease, serving as a valuable indicator for early risk screening [3]. In addition to organic causes, the development of ED is influenced by lifestyle habits, as well as metabolic and inflammatory pathways shared with benign prostatic hyperplasia [2, 4].

Patients with arterial hypertension are a primary focus in clinical practice. According to Kloner, in the hypertensive population aged 59 to 62 years, up to 67%–68% of patients exhibit some degree of erectile dysfunction, which is often more severe than in the general population [4, 5]. Elevated arterial pressure is directly linked to vascular damage and erectile dysfunction [6]. The pathophysiological mechanism primarily involves endothelial dysfunction secondary to oxidative stress, which impairs adequate vasodilation within the corpora cavernosa [5].

Antihypertensive therapy presents an additional challenge. While diuretics and beta‐blockers can impair erectile function and lead to treatment discontinuation, angiotensin receptor blockers (ARBs) generally have a neutral or beneficial effect [4, 6, 7]. However, significant differences may exist within the ARB class, driven by molecular structure and pleiotropic effects on endothelial function [8, 9]. In this paper, we report a case of a patient in whom switching from valsartan to olmesartan within a fixed‐dose combination was associated with a significant recovery of erectile function, as evidenced by an increase in the International Index of Erectile Function (IIEF‐5) score [10], despite the concurrent introduction of a low‐dose thiazide diuretic.

## 2 Case Presentation

### 2.1 Patient Demographics

A 53‐year‐old married man, a long‐time recreational athlete (cycling, strength training), was initially monitored for occasional elevations in blood pressure (BP) up to 140/90 mmHg and mixed hyperlipidaemia. Initial attempts at blood pressure control through lifestyle modifications (a low‐sodium diet and physical activity) did not achieve satisfactory control.

### 2.2 Treatment History

In December 2021, due to persistent BP values of 150/90 mmHg, therapy was initiated with perindopril (5 mg) and atorvastatin (40 mg). Due to inadequate blood pressure control (home measurements up to 160/97 mmHg), indapamide (1.5 mg) was briefly added; however, the patient reported poor adherence to treatment over the following 2 years. Between June and September 2025, the patient received a fixed‐dose combination of perindopril and amlodipine (5/5 mg, then 5/10 mg), which also failed to achieve target BP goals. Consequently, on 22 October 2025, therapy was switched to a fixed‐dose combination of valsartan and amlodipine (160/10 mg) once daily.

### 2.3 Clinical Problem and Diagnostic Workup

During 4 months of therapy with the valsartan/amlodipine combination, excellent blood pressure control was achieved (120/80–120/85 mmHg). However, within the first month of treatment, the patient noticed a significant development of erectile dysfunction (ED). Given that preserving sexual function was a priority, the patient expressed significant dissatisfaction with his quality of life and requested a change in therapy, even considering discontinuing treatment.

To rule out an endocrine cause of erectile dysfunction, the patient was referred for laboratory evaluation (total and free testosterone, SHBG), which yielded normal results (Table 1). In addition to documenting normal testosterone and SHBS levels, potential vascular, metabolic, psychological, lifestyle, and other medication‐related contributors to erectile dysfunction were thoroughly evaluated and considered non‐primary in this patient's presentation. Clinical suspicion focused primarily on a drug‐induced adverse effect probably associated with valsartan, given the clear temporal relationship between treatment initiation and symptom onset. Since calcium channel blockers such as amlodipine are classified as neutral regarding erectile dysfunction by Viigimaa et al. [6] and according to the results of the Treatment of Mild Hypertension Study (TOMHS) [11], the suspected cause of erectile dysfunction was primarily attributed to the valsartan component. This assumption was further supported by the patient's medical history, which showed that he had previously received combination therapy with perindopril and amlodipine (at doses of 5/5 mg and 5/10 mg) without experiencing erectile dysfunction, making amlodipine clinically unlikely as the primary cause.

**TABLE 1:** Endocrine laboratory workup results of the patient.

Parameter	Result	Reference range	Remark/Method
Total testosterone	14.49 nmol/L	8.67–38.14	LC–MS/MS
Free testosterone (%)	1.7%	1.5–2.2	Calculated (Vermeulen et al., 1999)
SHBG	43.7 nmol/L	13.5–71.4	CMIA Abbott

Although ARBs are generally protective, the observed temporal association indicates a possible idiosyncratic, individual lack of vascular benefit from valsartan in this patient's cavernous tissue. This discrepancy between the widely accepted pharmacological profile of the drug and the clinical outcome in this specific patient prompted a switch to olmesartan, a molecule with higher affinity for AT‐1 receptors. Although they belong to the same pharmacological class, potential molecular and pharmacodynamic differences between valsartan and olmesartan might explain the distinct clinical outcomes observed in our patient.

### 2.4 Intervention and Outcome

On 4 February 2026, a fixed‐dose combination of olmesartan (40 mg), amlodipine (5 mg), and hydrochlorothiazide (12.5 mg) was initiated at half a tablet daily. Under this regimen, the patient received 20 mg of olmesartan, 2.5 mg of amlodipine, and 6.25 mg of hydrochlorothiazide.

Following the change in therapy, blood pressure values remained within target ranges (104/75 to 123/81 mmHg in home measurements; 120/80 mmHg in the clinic). Notably, the patient reported a complete regression of erectile dysfunction symptoms (positive dechallenge). Symptomatic improvement in erectile function was subjectively noticed by patient at the one‐month follow‐up assessement, where the symptoms had completely disappeared. Symptoms did not recur after the introduction of olmesartan (successful substitution without relapse, despite retaining a lower dose of the amlodipine component). The probability of a causal relationship between valsartan and erectile dysfunction was assessed using the Naranjo algorithm [12], yielding a score of 7, which indicates a *probable* adverse drug reaction.

Improvement was evaluated in April 2026 using the International Index of Erectile Function (IIEF‐5). As the questionnaire is not officially validated in Croatian, an ad hoc translation was used for clinical purposes. The results were collected retrospectively: the patient rated his status on the previous therapy (valsartan) with a score of 12 (moderate ED), while his status at the two‐month follow‐up assessement after switching to olmesartan therapy was rated at 22 (normal function) (Table 2, Figure 1). The patient reported high satisfaction with the therapeutic outcome and an improved quality of life.

**TABLE 2:** Comparison of clinical parameters and erectile function during valsartan treatment and after switching to olmesartan.

Clinical parameter	Valsartan + Amlodipine (160 mg/10 mg)	Olmesartan^a^ + Amlodipine^a^ + HCTZ^a^ (20/2.5/6.25 mg)
Blood pressure (Office)	120/80 mmHg	120/80 mmHg
IIEF‐5 score	12 (Moderate ED)	22^b^ (Normal function)
Naranjo probability	7 (Probable ADR)	—
Clinical effect	Suspected drug‐induced ED	Significant recovery (Dechallenge)

^a^ The doses in the second column reflect the use of 1/2 tablet of the combination (40/5/12.5 mg).

^b^ The IIEF‐5 score improvement of 10 points occurred within 2 months of the therapy switch.

**FIGURE 1:**
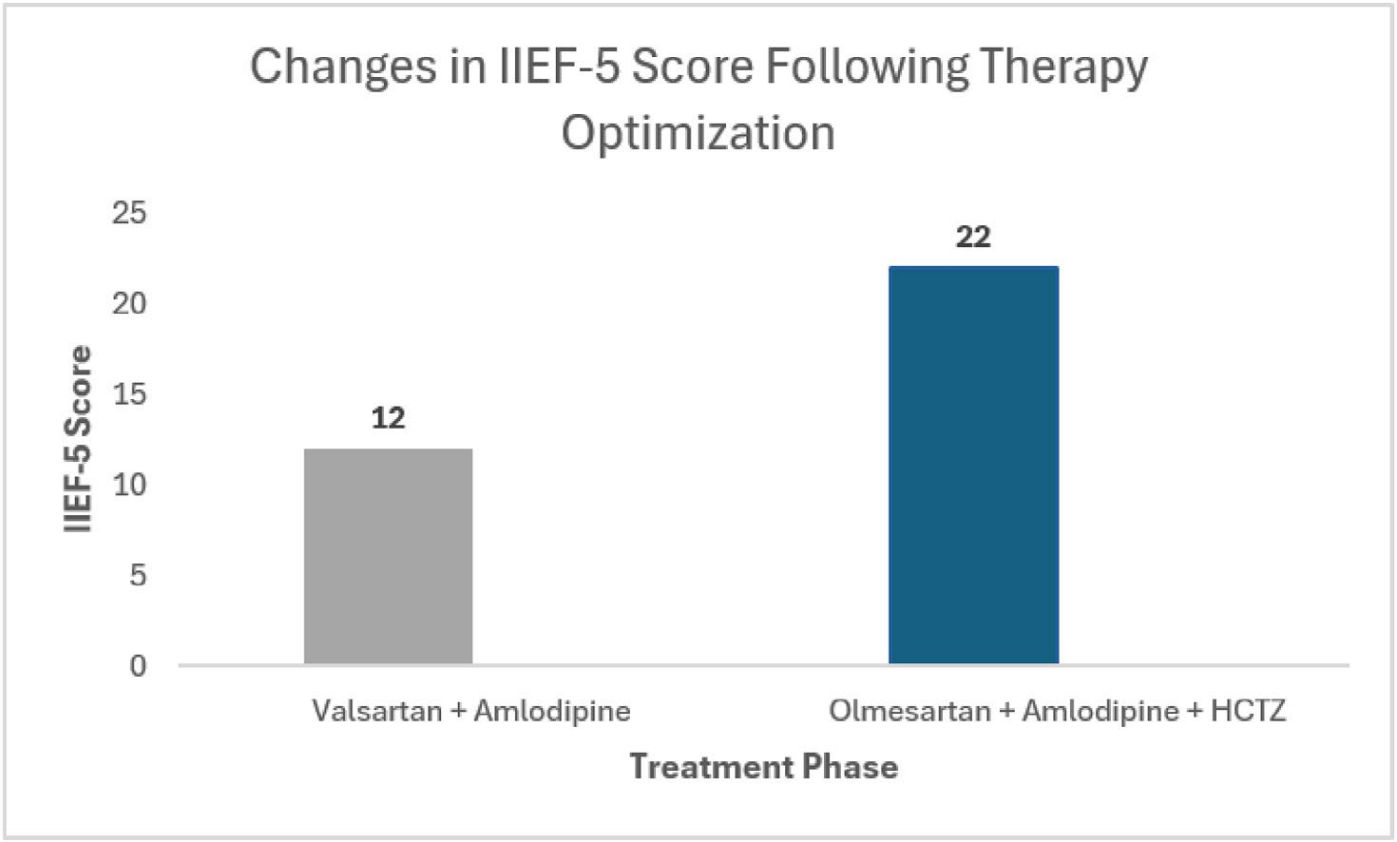
Comparison of patient‐reported International Index of Erectile Function (IIEF‐5) scores during the valsartan/amlodipine treatment phase and following the therapeutic switch to the olmesartan‐based triple fixed‐dose combination.

## 3 Discussion

Valsartan is a widely used angiotensin receptor blocker (ARB) indicated for the treatment of arterial hypertension, heart failure, and post‐myocardial infarction states. It is generally well tolerated as monotherapy. Clinical trials also indicate that fixed‐dose combinations of valsartan with hydrochlorothiazide or amlodipine at various doses effectively reduce aortic stiffness, central aortic pressure, and albuminuria, while improving endothelial function and quality of life. Notably, in these clinical trials, the combinations demonstrated a neutral metabolic profile and even had a positive effect on male erectile function [13, 14, 15]. Given the well‐documented protective effects of valsartan, the onset of erectile dysfunction (ED) in our patient was clinically unexpected. This observation challenges the assumption of uniform pharmacological class effects among ARBs and suggests potential molecular differences within the class.

In contrast, the negative impact of thiazide diuretics on erectile function is well established. Major studies, such as those by Chang et al. [16] and Curb et al. [17], confirm a strong correlation between diuretic use and the incidence of ED. Furthermore, the TOMHS [11] and Medical Research Council [18] trials recorded high treatment discontinuation rates due to diuretic‐induced side effects (17% and nearly 20%, respectively). However, it is important to note that these historical studies used substantially higher doses, such as chlorthalidone at 15 to 30 mg [11] or bendroflumethiazide at 5 mg twice daily [18]. In our case, administration of an ultra‐low dose of hydrochlorothiazide (only 6.25 mg) within a triple fixed‐dose combination provided excellent blood pressure control and was followed by a complete regression of ED. While it is possible that such low doses do not reach the pharmacological threshold required to compromise sexual function, the simultaneous adjustments in medication doses complicate a definitive attribution. By carefully evaluating the components, the substitution of the ARB remains a key area of interest, where potential molecular and pharmacodynamic differences between valsartan and olmesartan may provide a possible explanation for the observed clinical outcome.

Olmesartan medoxomil is a prodrug that undergoes rapid and complete conversion to its active form, olmesartan, during absorption from the gastrointestinal tract. Esterification with medoxomil is critical for enhancing the pharmacokinetic profile, significantly increasing the drug's bioavailability from an initial 6% to a clinically effective 28.6%. Another significant advantage of olmesartan is its minimal or absent inhibitory effect on the cytochrome P450 (CYP) enzyme system. Consequently, it has an exceptionally low potential for drug–drug interactions, which is of paramount importance in cases of polypharmacy among patients with comorbidities [19].

The clinical efficacy of olmesartan over other ARBs, including valsartan, is well documented in terms of robust blood pressure reduction [20]. Its specific pharmacodynamic properties are characterized by insurmountable antagonism, high affinity, and a slow dissociation rate from the AT‐1 receptor [21, 22]. At the molecular level, crystallographic analyses show that olmesartan engages in specific bipartite (double‐chain) binding within the receptor pocket, interacting simultaneously with the amino acid residues Tyr113 and His256 [23]. This distinct cooperativity stabilizes the receptor in its inactive state and mediates potent inverse agonism [22, 24] whereas valsartan binds to entirely different residues, primarily Lys199, and exhibits faster receptor dissociation [24]. Consequently, olmesartan may provide a more prolonged and stable blockade of the deleterious effects of angiotensin II (Ang II) in vivo [22].

Beyond receptor blockade, olmesartan has pronounced pleiotropic properties that directly protect the vascular wall, independent of blood pressure reduction. Evidence shows that olmesartan significantly reduces oxidative stress and microinflammation, normalizes the wall‐to‐lumen ratio of resistance vessels, and attenuates vascular remodeling [25, 26]. Hydrogen peroxide (H_2_O_2_), induced by the action of Ang II on the AT‐1 receptor, plays a pivotal role in arterial wall hypertrophy and endothelial dysfunction [27]. Because olmesartan may more effectively suppress Ang II‐induced intracellular free radical production due to its specific binding and sustained inverse agonism, this represents a plausible hypothesis for why endothelial dysfunction and subsequent suspected ED were resolved in our patient [25, 26, 27].

Definitive evidence of olmesartan's role in preserving sexual function comes primarily from animal models of hypertension. Research demonstrates that olmesartan, unlike some other fixed‐dose antihypertensive combinations, significantly decreases malondialdehyde concentrations (a marker of oxidative stress) in the corpus cavernosum, while directly upregulating messenger RNA (mRNA) levels for endothelial and neuronal nitric oxide synthase (eNOS and nNOS) in the penis [28]. A potential increase in the bioavailability of nitric oxide (NO) in the patient's cavernous tissue, driven by the stable molecular blockade of AT‐1 receptors and the antioxidant action of olmesartan, may have counteracted the vasoconstrictive potential of the low‐dose thiazide diuretic, serving as a potential explanation for the regression of erectile dysfunction following the withdrawal of valsartan [28].

### 3.1 Study Limitations

The primary limitation of this report is that it describes a single clinical case; therefore, any causal claims regarding the negative effects of valsartan or the benefits of olmesartan must be interpreted with caution and cannot be generalized without broader clinical trials. Furthermore, multiple therapeutic modifications occurred simultaneously: the ARB was switched, the amlodipine dose was reduced from 5 mg to 2.5 mg, and an ultra‐low dose of hydrochlorothiazide (6.25 mg) was introduced. Consequently, it is impossible to definitively isolate the therapeutic switch from valsartan to olmesartan as the sole driver of the improvement, even though the recovery occurred despite the addition of a thiazide. Finally, the use of a non‐validated Croatian translation of the IIEF‐5 questionnaire and its retrospective administration introduce potential recall bias, which must be acknowledged when interpreting the patient's reported scores. Indeed, this case underscores the need for future clinical trials to further investigate intra‐class differences among ARBs regarding sexual health.

## 4 Conclusion

This case report describes the complete regression of erectile dysfunction following a therapeutic transition from dual therapy with valsartan and amlodipine to a reduced‐dose triple fixed combination containing olmesartan, amlodipine, and hydrochlorothiazide. The clinical course highlights that ARBs should not be viewed as an entirely homogenous class regarding tissue‐specific or individual patient outcomes. In conclusion, the patient's erectile function significantly improved following the comprehensive adjustment of his antihypertensive management. Although this improvement strongly correlates with the treatment modification, the specific contribution of olmesartan cannot be isolated with certainty, given the concurrent reduction of the amlodipine dose and the introduction of ultra‐low‐dose hydrochlorotiazide. Ultimately, this case underscores the need for future clinical trials to further investigate intra‐class differences among ARBs regarding sexual health. For primary care physicians, timely recognition of suspected drug‐induced erectile dysfunction and implementing an intra‐class therapeutic switch represent a practical strategy to maintain patient adherence, prevent treatment discontinuation, and support long‐term cardiovascular prevention, thereby directly improving the patient's prognosis and quality of life.

## Author Contributions

**Mario Ćurković:** conceptualization, investigation, data curation, visualization, writing – original draft, project administration, resources, methodology, validation, supervision, formal analysis, funding acquisition. **Nika Ćurković:** investigation, writing – review and editing, software, formal analysis, data curation.

## Funding

The authors have nothing to report.

## Ethics Statement

This study was reviewed and approved by the Ethics Committee of Osijek‐Baranja County Health Center. Informed consent was obtained from the patient for the publication of this case report and any accompanying images or data.

## Consent

Written informed consent was obtained from the patient for the publication of this case report and any accompanying data or images. A copy of the written consent is available for review by the Editor‐in‐Chief of this journal upon request.

## Conflicts of Interest

The authors declare no conflicts of interest.

## Data Availability

The data that support the findings of this study are available from the corresponding author upon reasonable request.
